# Selenium-Substituted Hydroxyapatite/Biodegradable Polymer/Pamidronate Combined Scaffold for the Therapy of Bone Tumour

**DOI:** 10.3390/ijms160922205

**Published:** 2015-09-14

**Authors:** Ewa Oledzka, Marcin Sobczak, Joanna Kolmas, Grzegorz Nalecz-Jawecki

**Affiliations:** 1 Department of Inorganic and Analytical Chemistry, Faculty of Pharmacy with the Laboratory Medicine Division, Medical University of Warsaw, Banacha 1, Warsaw 02-097, Poland; E-Mails: marcin.sobczak@wp.pl (M.S.); joanna.kolmas@wum.edu.pl (J.K.); 2 Department of Environmental Health Science, Faculty of Pharmacy with the Laboratory Medicine Division, Medical University of Warsaw, Banacha 1,Warsaw 02-097, Poland; E-Mail: gnalecz@wum.edu.pl

**Keywords:** biodegradable polymers, bisphosphonates, hydroxyapatite, bone tumour, drug delivery systems, biocomposite

## Abstract

The present study evaluated a new concept of combined scaffolds as a promising bone replacement material for patients with a bone tumour or bone metastasis. The scaffolds were composed of hydroxyapatite doped with selenium ions and a biodegradable polymer (linear or branched), and contained an active substance—bisphosphonate. For this purpose, a series of biodegradable polyesters were synthesized through a ring-opening polymerization of ε-caprolactone or d,l-lactide in the presence of 2-hydroxyethyl methacrylate (HEMA) or hyperbranched 2,2-bis(hydroxymethyl)propionic acid polyester-16-hydroxyl (bis-MPA) initiators, substances often used in the synthesis of medical materials. The polymers were obtained with a high yield and a number-average molecular weight up to 45,300 (g/mol). The combined scaffolds were then manufactured by a direct compression of pre-synthesized hydroxyapatite doped with selenite or selenate ions, obtained polymer and pamidronate as a model drug. It was found that the kinetic release of the drug from the scaffolds tested *in vitro* under physiological conditions is strongly dependent on the physicochemical properties and average molecular weight of the polymers. Furthermore, there was good correlation with the hydrolytic biodegradation results of the scaffolds fabricated without drug. The preliminary findings suggest that the fabricated combined scaffolds could be effectively used for the sustained delivery of bioactive molecules at bone defect sites.

## 1. Introduction

In recent years, the trend for the development of biomaterials for implantology indicates the growing importance of composite materials based on polymers [1,2]. Nonetheless, the inimical mechanical properties of the polymeric materials, such as their mechanical strength, Young’s modulus and fracture toughness have limited their application as implants. It is possible to improve these properties and create composite materials when a modifying phase (e.g., hydroxyapatite (HA)) is applied to impart appropriate mechanical and biological properties. The application of the modifying phase provides the possibility of improving the poor mechanical properties of polymers (e.g., increasing the strength and fracture toughness while maintaining the Young’s modulus level at the module level of the bone tissue). A suitable modifier also enables the changing of the biological properties through giving them bioactivity. The idea of strengthening bioactive polymer composites by ceramic particles was introduced by Bonfield *et al.* [3], resulting in the mechanical similarity of these materials to a natural bone. On the other hand, the HA composite based on polyethylene (PE) was the first bioactive polymer-ceramic composite, applied for the production of a middle ear implant [4].

Aliphatic polyesters, e.g., poly(l-lactide) (PLA), poly(ε-caprolactone) (PCL) and copolymers of d,l-lactide (LA) and ε-caprolactone (CL), belong to the family of materials that make it possible to create a variety of implantable and/or controlled injection systems for the release of therapeutic agents, due to their biodegradability, biocompatibility and permeability [5,6]. These polymers are usually synthesized by the ring-opening polymerization (ROP) of LA, l,l-lactide (LLA) or ε-CL in the presence of multifunctional initiators containing nucleophile groups (hydroxyl or amine). Aside from acting as a true initiator, the compounds are used to build into the polymer chain as an end group in order to cause polymer functionalization (end-group functionalization). 2-hydroxyl ethyl methacrylate (HEMA) is often used in the synthesis of medical polymer materials (e.g., soft contact lenses), thermosetting coatings and adhesives [7,8], whereas poly-2,2-bis(methylol) propionic acid (bis-MPA) is a monomer unit used for the synthesis of a hyperbranched aliphatic polyester, e.g., Boltorn, a viable model system resembling dendrimers in drug delivery applications [9]. In recent years, Fréchet *et al.*, have developed bis-MPA-based dendrimers for nanomedical applications, including dendrimers for targeted positron emission tomography (PET) imaging, as well as for very efficient drug delivery carriers of doxorubicin [10,11]. Looking at these facts, the application of these compounds as initiators or macroinitiators for the ROP of cyclic esters appears very promising for further biomedical purposes. Recently, Martinelle *et al.*, used HEMA as an initiator of the enzymatic ROP of ω-pentadecatlone (PDL) and CL for the synthesis of well defined end-capping dimethacrylated polymers [12], while comb polymers were formed by a free radical polymerization of HEMA end-functionalized macromonomers of PCL in bulk [13]. On the other hand, HEMA-terminated oligo-l,l-lactide (OLLA) and oligo-d,l-lactide (ODLA) macromonomers with short OL(D)LA chain lengths were synthesized and copolymerized with HEMA to produce poly(HEMA-g-OL(D)LA)s for application as controlled release carriers for biomedical applications [14]. The design, synthesis and solution properties of new dendritic-linear hybrid macromolecules constructed from a high molecular weight PCL, initiated from the surface hydroxyl groups of bis-MPA in the presence of stannous 2-ethyl hexanoate (Sn(Oct)_2_) as a catalyst, has been described by Trollsås and colleagues [15]. The same authors described the synthesis of star PLA with well defined molecular architectures, also obtained from multifunctional dendritic initiators derived from 2,2′-bis(hydroxymethyl)propionic acid (bis-MPA) derivatives [16]. A series of branched polyesters consisting of PCL initiated from 32-, 24- and 8-hydroxy-functional cores based on bis-MPA and end-capped with methyl methacrylate were prepared by Claesson *et al.* [17]. Likewise, aliphatic AB2 functional polyesters were prepared by ROP of CL and LLA in the presence of the AB2 functional initiator bis-MPA and Sn(Oct)_2_ as a catalyst [18]. The authors found that in LLA polymerization, bis-MPA hydroxyl groups effectively initiated polymerization, but for CL polymerization, this process was dependent on the feed ratio of monomer to initiator and catalyst.

HA is known to be a biocompatible and bioactive material, and thus has been commonly used for the fabrication of highly porous, interconnected scaffolds and isotropic pore structures [19]. This material might be also applied as a coating for orthopaedic and dental implants, sustaining their surface properties and the bone-implant interface, leading to improvement in its osseointegration [20]. The development of new high-standard materials similar to natural bone apatite has recently attracted much attention [21]. One of the methods is the substitution of HA with various ions (*i.e.*, Mg^2+^, Na^+^, K^+^, Zn^2+^, F^−^, Cl^−^ or others), in order to enhance the biological, physicochemical and/or mechanical properties of HA. Selenium is one of the essential constituents of the human diet. Furthermore, it plays a significant role in diverse metabolic processes and is a constituent of selenoproteins as well as of glutathione peroxidase, the enzyme that protects cellular membranes against harmful agents [22,23]. Importantly, selenium may prevent carcinogenesis and inhibit the growth of tumour cells [24,25,26]; thus the use of HA doped with selenium ions (selenite or selenate) seems to be promising for further biomedical applications.

Bisphosphonates (BPs) are a chemical class of compounds in widespread use since the 1970s for the management of disorders of bone metabolism, associated with bone loss [27]. These compounds bind strongly to hydroxyapatite crystals, suppress osteoclast-mediated bone resorption, are retained for a long time in the skeleton and are excreted unmetabolized in urine [28]. One of the family of BPs is pamidronate (PAM)-alkyl-amino BP, considered the standard of care therapy for non-resectable skeletal metastases in human cancer patients [29]. PAM inhibits osteoclast migration and maturation when it binds to hydroxyapatite. Moreover, it normalizes serum calcium levels even in tumour-induced hypercalcaemia without detectable metastases [30].

In our previous work, the synthesis and characterization of composite (polyurethane/hydroxyapatite without selenium ions) clodronate delivery systems as potential candidates for application in the technology of implantation drug delivery carriers has been described [31]. In our current paper, we presented the preparation and characterization of the combined scaffolds composed of hydroxyapatite doped with selenium ions and a biodegradable polyester (linear or branched), and containing an active substance—pamidronate for potential application as a bone replacement material for patients with a bone tumour or bone metastasis. We anticipate that the prepared scaffolds could be promising as new biomaterials (bone implants) and controlled release drug delivery vehicles, especially those containing combinations of selenium and bisphosphonate. To the best of our knowledge, there is no literature report on the synthesis and characterization of such biomedical materials. To investigate our hypothesis, we have firstly synthesized and spectrally characterized linear and branched biodegradable polymers using HEMA or bis-MPA as an initiator or macroinitiator of ROP of LA or CL. The porous hydroxyapatite matrices were then manufactured by direct compression of hydroxyapatite doped with selenite or selenate ions, PAM and synthesized polymer. The structures and chemical compositions of the synthesized biomaterials were investigated and discussed based on the results obtained. Furthermore, the *in vitro* kinetic release of PAM from the scaffolds, as well as the hydrolytic degradation of the obtained materials, were studied.

## 2. Results and Discussion

Tissue-engineered bone substitute materials have been rapidly developing in recent years. One of the key components for successful functional tissue-engineered bone regeneration is the presence of scaffolds with optimal architecture and properties [19]. Therefore, in this study, combined scaffolds as a bone replacement material have been prepared and characterized.

### 2.1. Polyester Structures from 2-Hydroxyethyl Methacrylate (HEMA) and Hyperbranched 2,2-Bis(hydroxymethyl)propionic Acid Polyester-16-Hydroxyl (Bis-MPA) Initiated Ring Opening Polymerization (ROP)

**Scheme 1:**
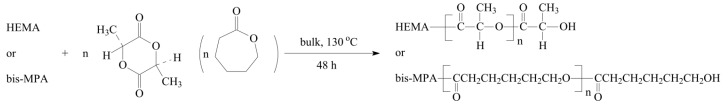
Synthesis of linear and branched polyesters using HEMA and bis-MPA as initiators.

In the first step of this study, linear and branched biodegradable polymers have been synthesized and characterized. HEMA or bis-MPA were used as the initiator or macroinitiator of ROP of CL or LA in bulk (Scheme 1).

Due to biomedical requirements, particularly the absence of toxic metal contaminants, polymerization has been carried out without any metal catalyst. In accordance with literature reports, we successfully obtained functionalized linear or branched polymers with HEMA or bis-MPA as the cores and hydroxyl end groups [7,11]. The polymerization in various molar ratios took place easily under the designated conditions. The results are shown in Table 1.

**Table 1:** Polymerization of d,l-lactide (LA) and ε-caprolactone (CL) initiated by HEMA and bis-MPA. Molecular characterization of the synthesized polymers.

Sample	I/M (mol/mol)	Yield (%)	*DP* ^c^	*DS* ^c^	*M*_n(*NMR*)_^c^	*M*_n(*GPC*)_^d^	*M*_W_/*M*_n_^d^
HEMA-PCL50	1:50	75	72	1.3	10,800	12,300	1.28
HEMA-PLA50	1:50	82	13	1.4	2800	3100	1.46
HEMA-PCL100	1:100	64	101	1.1	12,800	14,200	1.12
HEMA-PLA100	1:100	71	26	1.1	4300	4700	1.65
HEMA-PCL250	1:250	68	115	1.2	15,900	17,500	1.32
HEMA-PLA250	1:250	64	27	1.3	5200	5700	1.78
bis-MPA-PCL200	1:200	89	26	14.0	43,300	45,300	1.44
bis-MPA-PLA200	1:200	34	18	11.0	30,300	33,100	1.77

Reaction conditions: argon atmosphere, reaction temperature −130 °C; M = monomers; ε-caprolactone (CL) and d,l-lactide (LA), I = initiators; HEMA and bis-MPA, ^c^ *DP*—the average degree of polymerization determined by ^1^H NMR analysis, calculated based on the area ratio of the terminal methylene protons of PCL (3.64 ppm) or methine proton of PLA (4.35 ppm) to the internal methylene proton of PCL (4.12 ppm) or methine proton of PLA (5.11 ppm); *DS—*the average degree of substitution determined by ^1^H NMR analysis; calculated based on the peak areas or the methylene protons of PCL (3.64 ppm) or methine proton of PLA (4.35 ppm) and the methylene protons of HEMA (1.94 ppm) or methylene protons of bis-MPA (1.17–1.01 ppm); *M*_n(*NMR*)_ = 114.14 (or 144.13 g/mol) × *DP* × *DS* + *M*w (initiator: HEMA or bis-MPA—130.14 or 1749.79 g/mol); ^d^ The number-average molecular weight and polydispersity index were determined by gel permeation chromatography (GPC) analysis.

**Figure 1:**
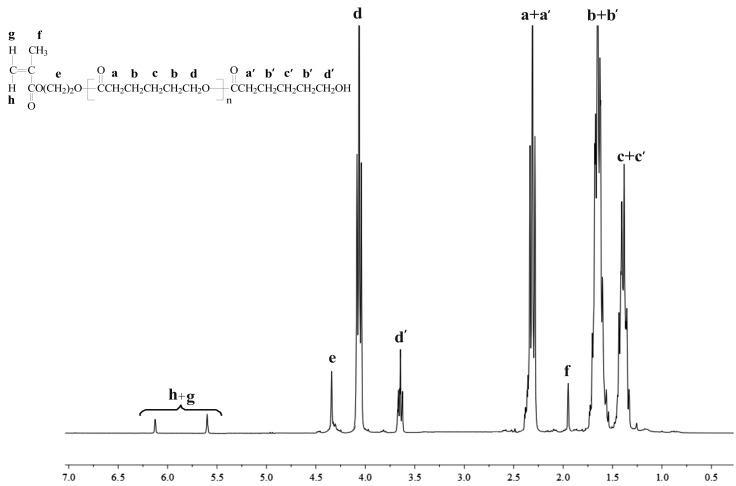
^1^H NMR spectra of HEMA-PCL100.

**Figure 2:**
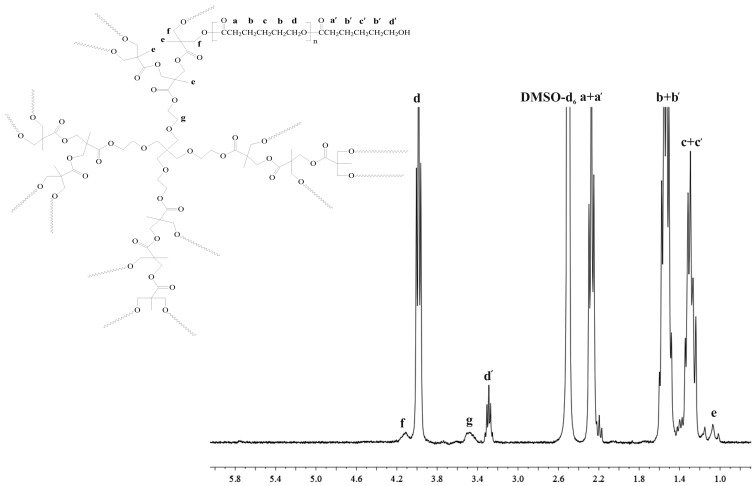
^1^H NMR spectra of bis-MPA-PCL200.

The structures of the products have been confirmed by ^1^H NMR spectra (Figure 1 and Figure 2).

The major resonance signals attributed to PCL and PLA chains as well as methylene or methine proton signals terminated by hydroxyl end groups can be easily observed on the spectra (Figure 1 and Figure 2 and Figure S2 and Figure S5). Importantly, the characteristic proton signals of HEMA (assigned as **h**, **g**, **e**, **f** in Figure 1) and bis-MPA moieties (assigned as **g**, **e**, **f** in Figure 2) were also detected with high resolution, indicating incorporation of HEMA or bis-MPA into macromolecule. ^1^H NMR analysis demonstrated that the initiators used successfully initiated ROP of CL or LA. An additional confirmation of the presence of HEMA or bis-MPA in the macromolecule is given by ^13^C NMR spectra (Figure S1, Figure S3, Figure S4 and Figure S6).

From the ^1^H NMR spectra of the obtained products, it was possible to determine the degree of polymerization (*DP*) and average degree of substitution (*DS*) and subsequently, their number-average molecular weight (*M*_n(NMR)_). The calculation results as well as gel permeation chromatography (GPC) data are shown in Table 1. The results reveal that one polymer arm can be attached to the HEMA initiator molecule, demonstrating that hydroxyl group of HEMA was an effective initiation centre of ROP of LA or CL. In the case of the bis-MPA initiator, there were a total of 16 initiating sites (–OH groups) in the bis-MPA molecule. The obtained results show that the *DS* of bis-MPA-PCL and bis-MPA-PLA was 11 and 14 (Table 1). According to the above calculation, not all the hydroxyl groups initiated ROP of CL or DLA, probably because of the change in density on the initiator surface and steric hindrance of the attached polymer chains. However, it was difficult to find unreacted hydroxyl end groups of bis-MPA moieties on ^1^H NMR spectra. Nonetheless, it was noted that *M*_n_ obtained by GPC is accurate to those calculated from ^1^H NMR analysis, and that polymeric materials with tolerable high yield and molecular weight distribution were prepared. One exception was the bis-MPA-PLA sample, obtained with a low yield of 34% (Table 1). Even if this reaction was repeated three times, the yield of the product was rather low. This fact might be explained by a slow reaction progress caused by solidification of the reaction mixture at the beginning of the process. Therefore, we decided that this synthesized polymeric sample would not be used for further studies.

As materials for medical and pharmaceutical applications must fulfill the relevant requirements (e.g., toxicity effect (PE) should be lower than 20 [32]), the synthesized polymeric products (HEMA-PCL, HEMA-PLA and bis-MPA-PCL) were subjected to cytotoxicity tests. The luminescent bacteria *V. fischeri* and ciliated protozoa *S. ambiguum* were used to perform cytotoxicity assays. In the Spirotox test, it has been found that the polymeric extracts (four different polymer concentrations) have zero values (Table 2). In the Microtox test, the polymeric extracts were also tested at four different polymer concentrations (0.8, 0.4, 0.2 and 0.1 mg/mL). From the obtained results, it was found that the samples with a higher polymer concentration (0.8 mg/mL for HEMA-PCL and HEMA-PLA) were slightly toxic to bacteria. However, after a two-fold extract dilution, the PE dropped below 20, thus we can consider the samples non-toxic for bacteria. The bis-MPA-PCL sample did not caused any negative reaction for the testing organism (Table 2).

**Table 2:** The cytotoxicity of the obtained polymeric samples.

Cytotoxicity Tests	Microtox^®^ 15 min-PE ^a^				Spirotox 24 h-PE ^a^			
Concentration (mg·mL^−1^)	0.8	0.4	0.2	0.1	1.0	0.5	0.25	0.125
HEMA-PCL100	22 ± 2	12 ± 1	3 ± 2	1 ± 1	0	0	0	0
HEMA-PLA100	26 ± 5	14 ± 1	2 ± 2	3 ± 3	0	0	0	0
Bis-MPA-PCL200	1 ± 1	8 ± 3	3 ± 3	2 ± 1	0	0	0	0

^a^ Percent of toxic effect (luminescence decreasing in the Microtox test and deformation in the Spirotox test).

### 2.2. In Vitro Pamidronate (PAM) Release Characteristics of the Manufactured Scaffolds

**Figure 3:**
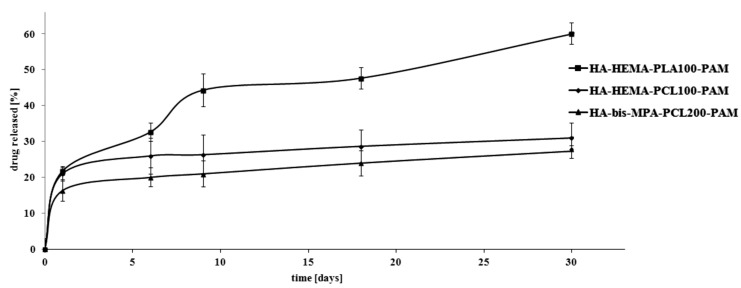
Release profile of pamidronate (PAM) from the manufactured combined scaffolds (pH 7.4 ± 0.05).

The combined scaffolds were manufactured in pellet form by a direct compression of selenium-substituted hydroxyapatite, synthesized polymer and PAM. HA doped with selenite ion has been selected for the preparation of HA-HEMA-PCL100-PAM and HA-HEMA-PLA100-PAM combined scaffolds. For the manufacturing of HA-bis-MPA-PCL200-PAM scaffold, HA doped with selenate ion has been used, by contrast. Figure 3 shows the *in vitro* release profile of PAM from the prepared combined scaffolds.

The total percentage of the released PAM from the HA-HEMA-PLA100-PAM, HA-HEMA-PCL100-PAM and HA-bis-MPA-PCL200-PAM scaffolds was approximately 60%, 31% and about 27% after 30 days of incubation. We assume that the above results could be affected by a higher degree of crystallinity and hydrophobicity in PCL than PLA. The mass percentage of PAM released from the HA-HEMA-PLA100-PAM scaffold was about 33% after six days of degradation. After this point, a significant rate in PAM release was observed: 44% of PAM was released after nine days, followed by a sustained release of the remaining PAM over the next 30 days. This phenomenon might be explained by a drug release from the amorphous polymer phase early on, and then from the crystalline polymer phase. In comparison with HA-HEMA-PLA100-PAM scaffold, the release profiles of HA-HEMA-PCL100-PAM and HA-bis-MPA-PCL200-PAM appear similar, but lower amounts of PAM are released over the study period: 21% and 26% PAM was released after nine days of incubation without evident collapse in the profile. We assume that this release behavior may be due to the PCL layer on the surface of the doped HA, which allowed a slow release of PAM through the HA pores.

**Figure 4:**
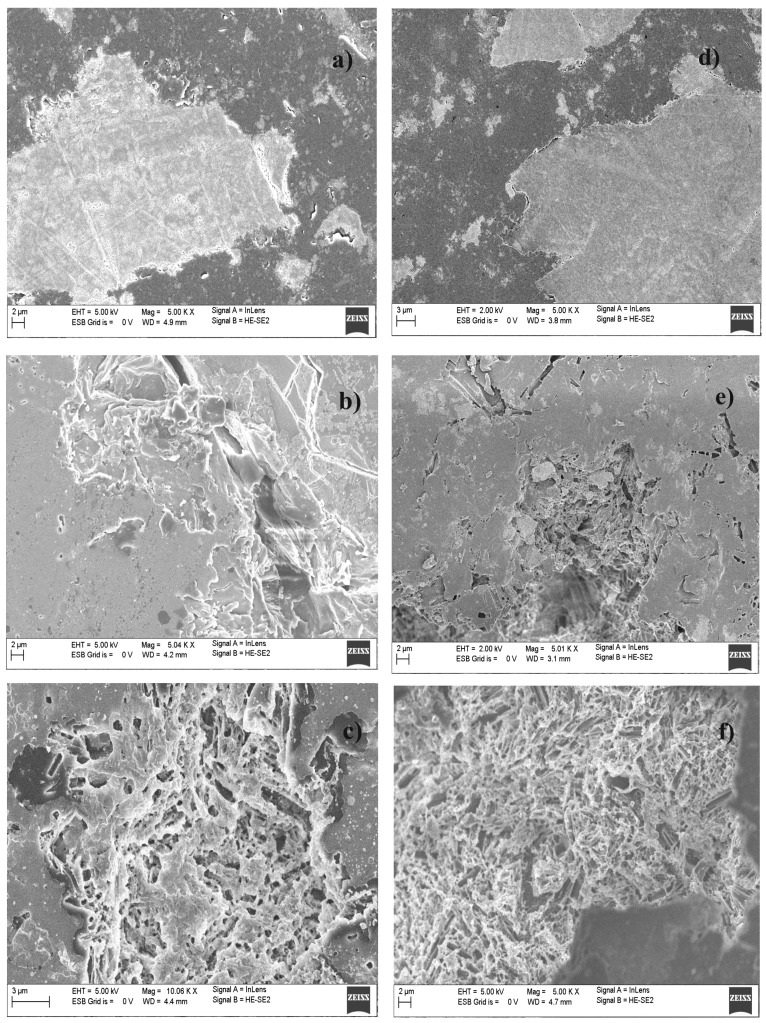
SEM micrographs of HA-HEMA-PCL100-PAM: before immersion in PBS (**a**); after immersion in PBS for nine days (**b**) and 30 days (**c**) and HA-bis-MPA-PCL200-PAM: before immersion in PBS (**d**); after immersion in PBS for nine days (**e**) and 30 days (**f**).

The representative SEM images of the surface of the combined scaffolds of the HA-HEMA-PCL100-PAM and HA-bis-MPA-PCL200-PAM (before and after incubation in PBS) are presented in Figure 4 (unfortunately, the scaffolds composed of PLA (pellet form) disintegrated into powder after seven days of incubation in PBS, thus it was impossible to perform SEM analysis of their surface. Apparently, this occurrence may be explained by hydrophilic properties of PLA *versus* PCL). The morphology of the scaffolds surface before degradation (Figure 4a,d) reveals a smooth appearance with an evident particulate phase. This particulate phase may result from the PAM incorporated in the polymeric matrix and HA phase. The surface of both scaffolds was found to be free from any irregularities such as cracking and delamination. The SEM images of the scaffolds in Figure 4b,c,e,f were obtained following their nine and 30-days incubation in PBS. The voids and holes observed on the surface were regions previously occupied by a PAM particle that was partially released from the scaffold; after that process, the surface becomes cracked and porous. This indicated that the drug release was via the dissolution of PAM from the combined scaffolds surface and a bulk erosion of the polymer and HA. Due to the approximate percentage of PAM released after nine and 30-days incubation in PBS, there was no significant and visible difference in the SEM images of the surface of the HA-HEMA-PCL100-PAM (Figure 4b,c) compared to the HA-bis-MPA-PCL200-PAM (Figure 4e,f).

### 2.3. Hydrolytic Degradation Behavior of the Manufactured Scaffolds

**Figure 5:**
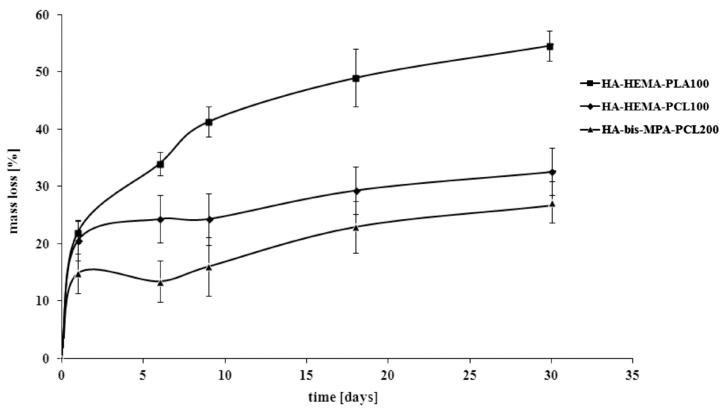
Effect of hydrolytic degradation time on the weight loss (*WL*) of the manufactured combined scaffolds.

The drug release characteristic was correlated with the hydrolytic degradation results of the obtained combined scaffolds by monitoring their weight loss (*WL*). The results are shown in Figure 5.

**Figure 6:**
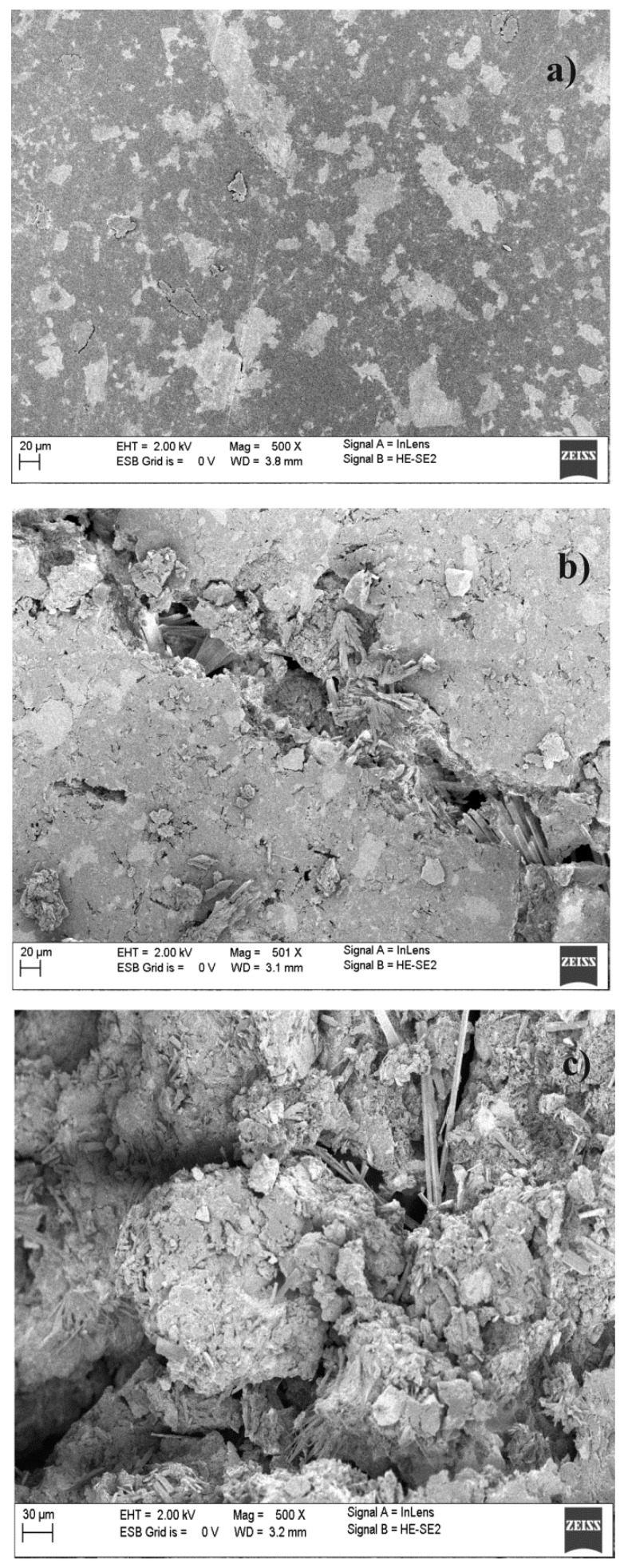
SEM micrographs of HA-bis-MPA-PCL200: before hydrolytic degradation (**a**); after hydrolytic degradation for (**b**) nine days and (**c**) 30 days.

The *WL* values of the HA-HEMA-PLA100 scaffold reached about 55% after 30 days of degradation, whereas 33% and 27% were achieved in the same study time for HA-HEMA-PCL100 and HA-bis-MPA-PCL200, respectively. The scaffold based on PLA was characterized by a lower hydrolytic stability as opposed to the scaffolds based on PCL; thus, the *WL* in these two cases was lower. These results were well correlated with the percentage of PAM released, thereby confirming the dependence of the physicochemical properties as well as the average molecular weight of the synthesized polymers on the drug release profile. The hydrolytic degradation characteristic of the surface of the manufactured combined scaffolds has also been characterized by SEM analysis. As an example, the scanning electron microscopic images of the HA-bis-MPA-PCL200 scaffold, in their original state as well as after 30 days of degradation, are shown in Figure 6. In comparison to the scaffold before degradation (Figure 6a), the surface of the scaffold after 30 days exhibited a well developed open pore structure and severe cracking all over the surface, indicating significant oxidative and hydrolytic damages (Figure 6b,c).

In our previous work [31], new composite bisphosphonate delivery systems were obtained from polyurethanes, nanocrystalline hydroxyapatite and clodronate as good potential candidates for application in the technology of implantation drug delivery carriers. The mechanical properties, as well as toxicity of the obtained biomaterials were determined. The *in vitro* drug release characteristic showed that the rate of clodronate released was directly dependent upon the nature of the obtained polyurethanes and the porosity of the composites. Moreover, a good correlation was found between the changes in the mechanical properties and the *M*_v_ of the obtained polyurethanes and the *in vitro* degradation results of the produced composites (for instance, the changes in the *M*_v_ for the obtained polyurethanes were around 4.7%–6.0% after four weeks and 7.2%–11.4% after eight weeks of degradation). In this work, the combined scaffolds, composed of the synthesized hydroxyapatite doped with selenium ions, biodegradable polyester (linear or branched) and containing PAM as a model drug, were successfully developed as an excellent drug delivery platform for use as a bone replacement material. It has been found that the total percentage of the released PAM from the appropriate scaffolds was approximately 60% (material based on PLA), 31% and about 27% (material based on PCL) after 30 days of degradation, and the results were affected by physicochemical properties and the number-average molecular weight of the synthesized polymeric matrices. The results obtained in these two works confirmed that the nature of the polymeric materials has a significant influence on the drug release and hydrolytic degradation characteristic (higher hydrolytic stability of polyurethanes compared to PLA or PCL homopolymers). Furthermore, the biomedical composites based on polyurethanes are likely promising materials for medium- or long-term implantation bisphosphonate delivery systems. For comparison, the scaffolds obtained in this work might instead find practical applications as short-term implantation bisphosphonate delivery systems.

In our current work, hydroxyapatite doped with selenium ions and containing PAM has been used for manufacturing the combination scaffolds as new bone replacement materials due to the synergistic action of selenium and bisphosphonates. Since bisphosphonates exhibit a strong affinity to nonocrystalline hydroxyapatites (they may strongly adsorb on the apatitic surface by an ion-exchange mechanism between phosphonate groups from bisphosphonates and phosphate ions from hydroxyapatite), application of the synthesized selenium doped hydroxyapatite might also yield promising results. Therefore, the studies on the correlation of the composites structure and the drug release kinetics under different pH conditions are currently underway and the results will be presented in our next paper.

## 3. Experimental Section

### 3.1. Materials

ε-Caprolactone (2-oxepanone, ≥99.0%, Aldrich Co., Poznan, Poland) was dried and distilled before use over CaH_2_ at reduced pressure. d,l-Lactide (3,6-Dimethyl-1,4-dioxane-2,5-dione, 98.0%, Aldrich Co., Poznan, Poland) was recrystallized from dried ethyl acetate in a dry nitrogen atmosphere and then thoroughly dried in vacuum before use. 2-hydroxyethyl methacrylate (HEMA, 97.0%, Aldrich Co., Poznan, Poland) and hyperbranched 2,2-bis(hydroxymethyl)propionic acid polyester-16-hydroxyl, generation 2 (bis-MPA, ≥97%, Aldrich Co., Poznan, Poland) were thoroughly dried in a vacuum. Disodium pamidronate hydrate (PAM, purity >97.0%, TCI EUROPE N.V., Zwijndrecht, Belgium) was dried before use. Dichloromethane (anhydrous, ≥99.8%, Avantor Performance Materials S.A., Gliwice, Poland), dimethylformamide (DMF, anhydrous, 99.8%, Avantor Performance Materials S.A., Gliwice, Poland), hexane (anhydrous, 99.8%, Avantor Performance Materials S.A., Gliwice, Poland) and diethyl ether (anhydrous, 99.8%, Avantor Performance Materials S.A., Gliwice, Poland) were used as received. Phosphate buffer solution (pH 7.4 ± 0.05, 0.1 M (potassium dihydrogen phosphate/disodium hydrogen phosphate), 20 °C, Avantor Performance Materials S.A., Gliwice, Poland) was also used as received.

### 3.2. Polymerization Procedure

The polymeric materials were prepared using different molar ratios of initiator (HEMA or bis-MPA) to monomers (ε-caprolactone (CL) or d,l-lactide (LA)). The initiator/monomer feed ratios for the obtained polymers were: 1/50; 1/100; 1/200 and 1/250 (mol/mol) denoted as HEMA-PCL50; HEMA-PLA50; HEMA-PCL100; HEMA-PLA100; HEMA-PCL200; HEMA-PLA200, bis-MPA-PCL250 and bis-MPA-PLA250; respectively, where HEMA = 2-hydroxyethyl methacrylate; bis-MPA = hyperbranched bis-MPA polyester-16-hydroxyl; PLA = poly(l-lactide) and PCL = poly(ε-caprolactone). For each polymerization, dry HEMA or bis-MPA and LA or CL were accurately weighted and introduced into 50 or 200 mL polymerization tubes. The tube was then connected to a Schlenk line, where exhausting-refilling processes were repeated three times. The tube was immersed into an oil bath at 130 °C under argon atmosphere for 48 h. After an appropriate time, the reaction products were cooled down, dissolved in dry CH_2_Cl_2_ or DMF, precipitated twice from a cold mixture hexane/diethyl ether and dried under vacuum for 72 h.

#### 3.2.1. NMR Data for HEMA-PCL100

^1^H NMR (CDCl_3_, 300 MHz, δ_H_, ppm); 6.12 and 5.62 (s, C**H_2_**=C(CH_3_)C(O)(CH_2_)_2_O of HEMA (**g** + **h**)), 4.41 (t, CH_2_=C(CH_3_)C(O)(C**H_2_**)_2_O of HEMA (**e**)), 4.12 (t, –C**H_2_**O– of PCL (**d**)), 3.64 (t, –C**H_2_**OH, end group of PCL (**d′**)), 2.33 (t, –CH_2_C**H_2_**C(O)– of PCL (**a** + **a′**)), 1.94 (s, CH_2_=C(C**H_3_**)C(O)(CH_2_)_2_O of HEMA (**f**)), 1.77 (m, –C**H_2_**CH_2_C(O)– of PCL (**b** + **b′**)) and 1.48 (m, –CH_2_C**H_2_**CH_2_– of PCL (**c** + **c′**)), (Figure 1).

^13^C NMR (CDCl_3_, 300 MHz, δ, ppm); 175.6 (**e′**), 173.6 (**e** + **i**), 137.4 (**h**), 126.3 (**g**), 67.3 (**j**), 64.2 (**d**), 62.6 (**d′**), 34.2 (**a** + **a′**), 28.4 (**b** + **b′**), 25.6 (**c** + **c′**), 24.7 (**k**), (Figure S1).

#### 3.2.2. NMR Data for HEMA-PLA100

^1^H NMR (CDCl_3_, 300 MHz, δ_H_, ppm); 6.11 and 5.60 (s, C**H_2_**=C(CH_3_)C(O)(CH_2_)_2_O of HEMA (**e** + **f**)), 5.11 (q, –C**H**(CH_3_)– of PLA (**a**)), 4.46 (t, CH_2_=C(CH_3_)C(O)(C**H_2_**)_2_O of HEMA (**c**)), 4.35 (q, –C**H**(CH_3_), end group of PLA (**a′**)), 1.90 (s, CH_2_=C(C**H_3_**)C(O)(CH_2_)_2_O of HEMA (**d**)), 1.67–1.55 (m, –C**H_3_** of PLA (**b** + **b′**)), (Figure S2).

^13^C NMR (DMSO-*d*_6_, 300 MHz, δ, ppm); 174.4 (**c′**), 168.4 (**c**), 136.2 (**f**), 125.1 (**g**), 72.3 (**a**), 68.6 (**a′**), 66.1 (**d**), 21.1 (**e**), 16.7 (**b′**), 15.1 (**b**), (Figure S3).

#### 3.2.3. NMR Data for Bis-MPA-PCL200

^1^H NMR (DMSO-*d*_6_, 300 MHz, δ_H_, ppm); 4.13 (m, –C(O)C(CH_3_)C**H_2_**O of bis-MPA (**f**)), 3.98 (t, –C**H_2_**O– of PCL (**d**)), 3.49 (m, –OC**H_2_**C**H_2_**O– of bis-MPA (**g**)), 3.35 (t, –C**H_2_**OH, end group of PCL (**d′**)), 2.27 (t, –CH_2_C**H_2_**C(O)– of PCL (**a** + **a′**)), 1.53 (m, –C**H_2_**CH_2_C(O)– of PCL (**b** + **b′**)), 1.31 (m, –CH_2_C**H_2_**CH_2_– of PCL (**c** + **c′**)), 1.17–1.01 (m, C**H_3_** groups of bis-MPA (**e**)), (Figure 2).

^13^C NMR (DMSO-*d*_6_, 300 MHz, δ_H_, ppm); 174.8 (**f**), 173.5 (**e′**), 172.9 (**e**), 65.5 (**g**), 63.4 (**d**), 60.5 (**d′**), 51.2 (**h**), 49.3 (**i**), 33.9 (**a** + **a′**), 28.0 (**b** + **b′**), 24.3 (**c** + **c′**), 17.5 (**j**), (Figure S4).

#### 3.2.4. NMR Data for Bis-MPA-PLA200

^1^H NMR (DMSO-*d*_6_, 300 MHz, δ_H_, ppm); 5.08–5.20 (q, –C**H**(CH_3_)– of PLA (**a**)), 4.38 (q, –C**H**(CH_3_), end group of PLA (**a′**)), 4.14 (m, –C(O)C(CH_3_)C**H_2_**O of bis-MPA (**d**)), 3.40 (m, –OCH_2_CH_2_O– of bis-MPA (**e**)), 1.69–1.58 (m, –C**H_3_** of PLA (**b** + **b′**)), 1.19–1.01 (m, C**H_3_** groups of bis-MPA (**c**)), (Figure S5).

^13^C NMR (DMSO-*d*_6_, 300 MHz, δ_H_, ppm); 174.3 (**h**), 171.2 (**c′**), 169.1 (**c**), 68.6 (**a**), 65.5 (**a′**), 64.4 (**d**), 51.8 (**f**), 49.7 (**g**), 18.4 (**b′**), 17.7 (**e**), 16.4 (**b**), (Figure S6).

### 3.3. Toxicity Assays

A Microtox^®^ assay with the luminescent bacteria *Vibrio fischeri* was performed with the lyophilized bacteria purchased from Modern Water (New Castle, PA, USA). The test was performed using disposable glass cuvettes. As a diluent and a control, 2% NaCl containing a 20 mM Tris buffer (pH 7.4) was used. Samples were incubated at 15 °C for 15 and 30 min and the light output of the samples was recorded with a Microtox^®^ M500 analyser. One millilitre of the extract refers to 0.8, 0.4, 0.2 and 0.1 mg of the polymer.

A Spirotox test with the ciliate protozoan *Spirostomum ambiguum* was performed according to the standard protocol [33]. The test was carried out in 24 disposable, polystyrene microplate wells. As a diluent and a control, Tyrode’s solution was used. Ten organisms were added to each of the microplate wells. The samples were incubated in the dark at 25 °C for 24 h. Following this, the test responses, *i.e.*, the different deformations of the cell and lethal responses, were observed with the use of a dissection microscope. Then, 10 mg of the polymer was extracted with 10 mL of Tyrode’s solution at 37 °C for 24 h. Prior to the toxicity test, the extract was neutralized with 0.1 M NaOH. Then, 1 mL of 0.4 M Tris (pH 7.4) was added and the Tyrode’s solution was poured up to 20 mL. One millilitre of the extract refers to 1.0, 0.5, 0.25 and 0.125 mg of the polymer. All samples were run in triplicate.

### 3.4. Manufacturing of Combination Scaffolds for in Vitro and Hydrolytic Biodegradation Studies

The synthesis and characterization of selenium-substituted hydroxyapatites containing selenate SeO_4_^2−^ or selenite SeO_3_^2−^ ions was reported in our previous paper [21]. The obtained combination scaffolds for both *in vitro* release and hydrolytic degradation studies have been manufactured in pellet form. For *in vitro* release studies, the pellets (ø 13 mm, flat-edged, 300 mg) composed of selenium-substituted hydroxyapatite (35%, *w*/*w*), synthesized biodegradable polymer (60%, *w*/*w*) and containing PAM (5%, *w*/*w*) were prepared via direct compression (98 kN) using a hydraulic press (Specac, London, UK). Prior to the compression, powder scaffolds were prepared via a physical mixing of the materials in the mortar.

For the hydrolytic biodegradation, the pellets (ø 13 mm, flat-edged, 300 mg) composed of selenium-substituted hydroxyapatite (40%, *w*/*w*) and synthesized biodegradable polymer (60%, *w*/*w*) have been manufactured in the same way as for release studies.

### 3.5. In Vitro PAM Release Studies form the Manufactured Composites

The *in vitro* release study of PAM was performed to measure the concentration of drug released at pH 7.4 ± 0.05 (0.1 M phosphate buffer solution). All experiments were carried out in triplicate; about 300 mg of dried combination scaffold (one pellet) was immersed in 10 mL buffer solutions (pH 7.4 ± 0.05) and incubated at 37 °C with continuous orbital rotation at 50 cycles/min. At predetermined time intervals, 10 mL samples were withdrawn from the release medium and then analysed by means of HPLC CAD (charged aerosol detector).

### 3.6. Hydrolytic Degradation

The hydrolytic degradation of the obtained scaffolds (one pellet of HA-HEMA-PLA100, HA-HEMA-PCL100 or HA-bis-MPA-PCL200) was performed in a 10 mL phosphate buffer solution (pH 7.4 ± 0.05) at 37 °C for 30 days. Following hydrolysis, the material samples were washed intensively with distilled water to remove any residual buffer solution, followed by drying under reduced pressure for five days. The degradation rates were estimated by weight loss (*WL*, (%)), calculated with the following equation:

*WL* (%) = 100 × (*W*_0_ − *W*_t_)/*W*_0_ (1)

where *W*_0_ = initial weight and *W*_t_ = weight after degradation.

### 3.7. Measurements

The polymerization products were characterized in the CDCl_3_ or DMSO-*d*_6_ solution by means of ^1^H and ^13^C NMR (Varian 300 MHz, LabX, Midland, ON, Canada).

Number-average molar masses (*M*_n_) and polydispersity indexes (*M*_W_/*M*_n_) of the obtained products were determined using an Agilent 1100 isocratic pump and a set of two PLgel 5 m mixed-C columns. An Optilab rEX Wyatt interferometric refractometer and a DOWN EOS Wyatt (Wyatt Technology Corporation, Santa Barbara, CA, USA) laser photometer were applied as detectors in a series. ASTRA 4.90.07 software (Wyatt Technology Corporation) was used for data collection and processing. Dichloromethane was used as an eluent at a flow rate of 0.8 mL·min^−1^ and polystyrene as a standard.

The quantity of released pamidronate was analysed by means of HPLC CAD using the UHPLC Dionex Ultimate 3000 analytical system with a CAD detector. Chromatographic separations were carried out using the Luna C8 column (250 × 4.6 mm, 5 μm). The calibration curve was obtained by the analysis of different concentrations of PAM in PBS solution (0.15–2.00 mg/mL). The analytical method was validated by the Pharmaceutical Research Institute in Poland.

The surface morphologies (before and after degradation) were studied by scanning electron microscope (SEM, Merlin, Zeiss, Jena, Germany) and then compared with the initial morphologies.

## 4. Conclusions

The combined scaffolds, composed of the synthesized hydroxyapatite doped with selenium ions and biodegradable polymer (linear or branched) and containing PAM as a model drug, were successfully developed as an excellent drug delivery platform for use as bone replacement material. In order to prepare biodegradable polymers, bulk ROP of CL and LA initiated with HEMA and bis-MPA was carried out. The structure and physicochemical properties of the obtained products were confirmed by ^1^H, ^13^C NMR and GPC methods. Furthermore, the cytotoxicity of the synthesized polymers was evaluated with a bacterial luminescence test and protozoan assay, which showed that the obtained polymers are not cytotoxic. *In vitro* results showed that the total percentage of the released PAM from the appropriate scaffolds was approximately 60% (material based on PLA), 31% and about 27% (material based on PCL), and the results were affected by physicochemical properties and the number-average molecular weight of the synthesized polymeric matrices. Consequently, these newly developed combined scaffolds might be applied as a promising material for patients with bone tumors or bone metastasis (jawbone).

## Supplementary Materials

Supplementary materials can be found at http://www.mdpi.com/1422-0067/16/09/22205/s1.
