# Supplementary material for: Selenium-Substituted Hydroxyapatite/Biodegradable Polymer/Pamidronate Combined Scaffold for the Therapy of Bone Tumour
Source: Int J Mol Sci. 2015 Sep 14;16(9):22205–22. doi: 10.3390/ijms160922205 (PMC4613304; doi:10.3390/ijms160922205)
Supplement: Supplementary file 1 [file ijms-16-22205-s001.pdf]

Supplementary Information

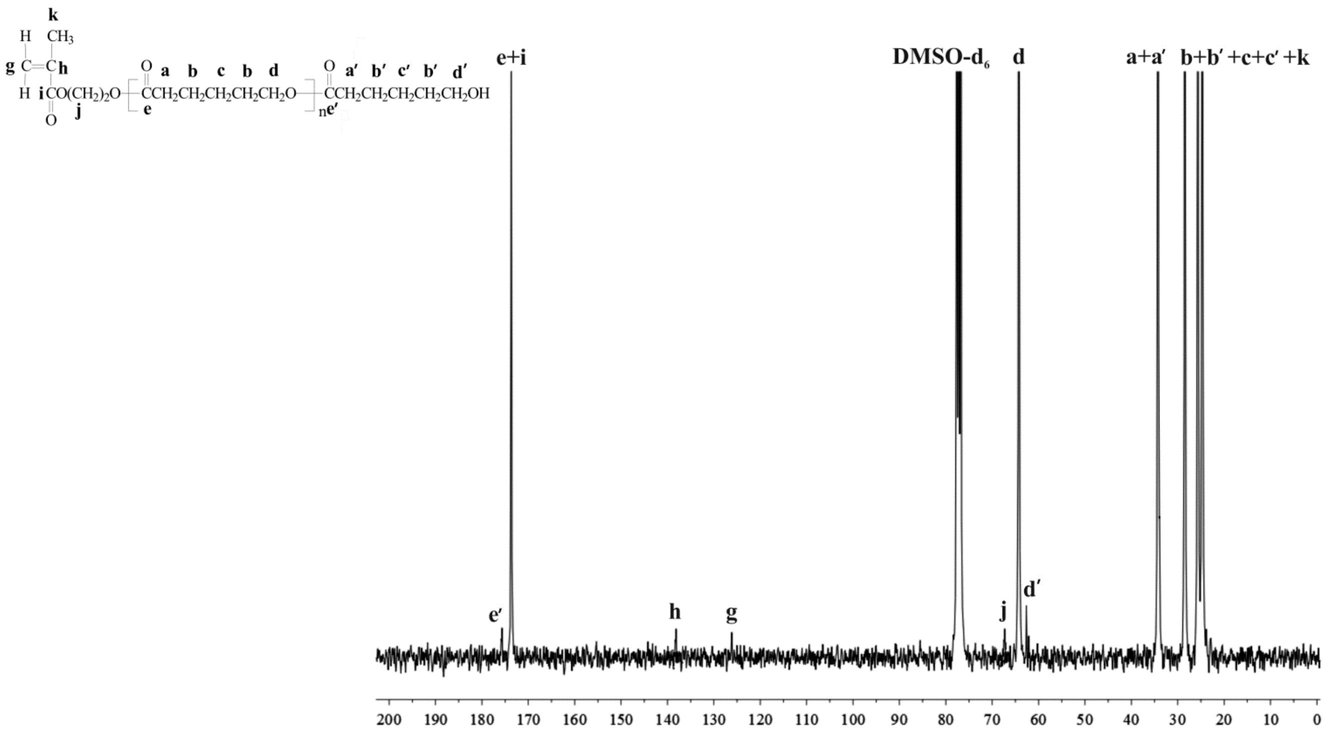

Figure S1. ^{13}C NMR spectrum of the HEMA-PCL100.

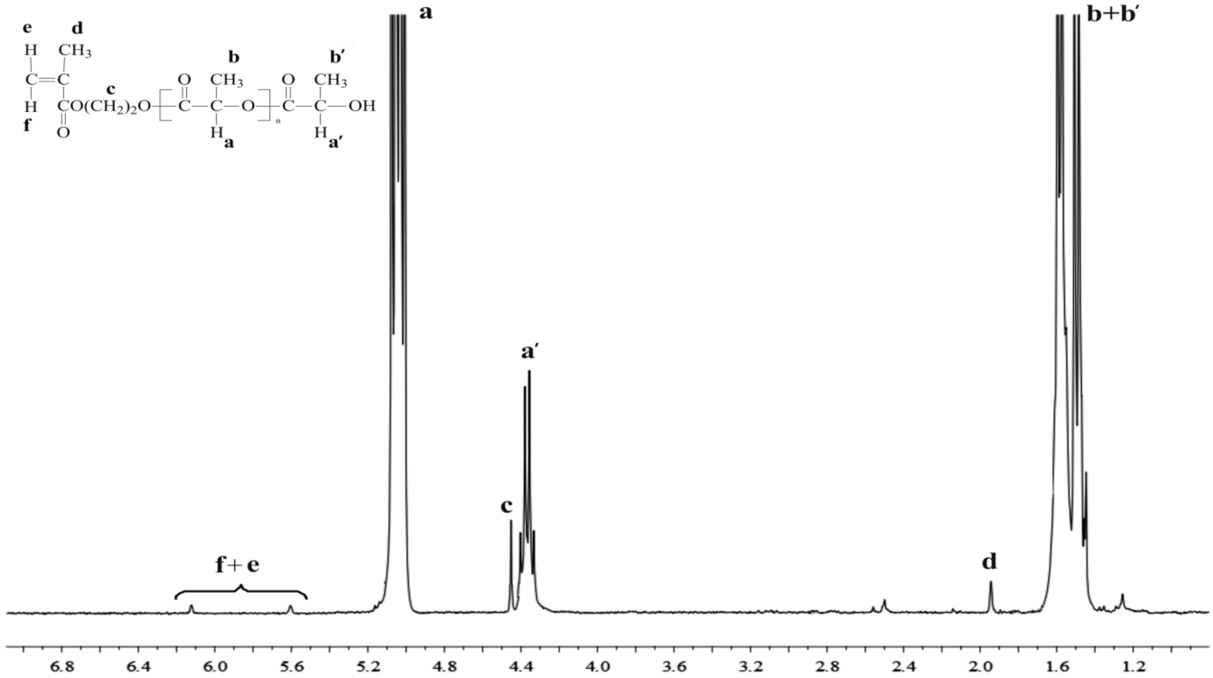

Figure S2. ^{1}H NMR spectrum of the HEMA-PLA100.

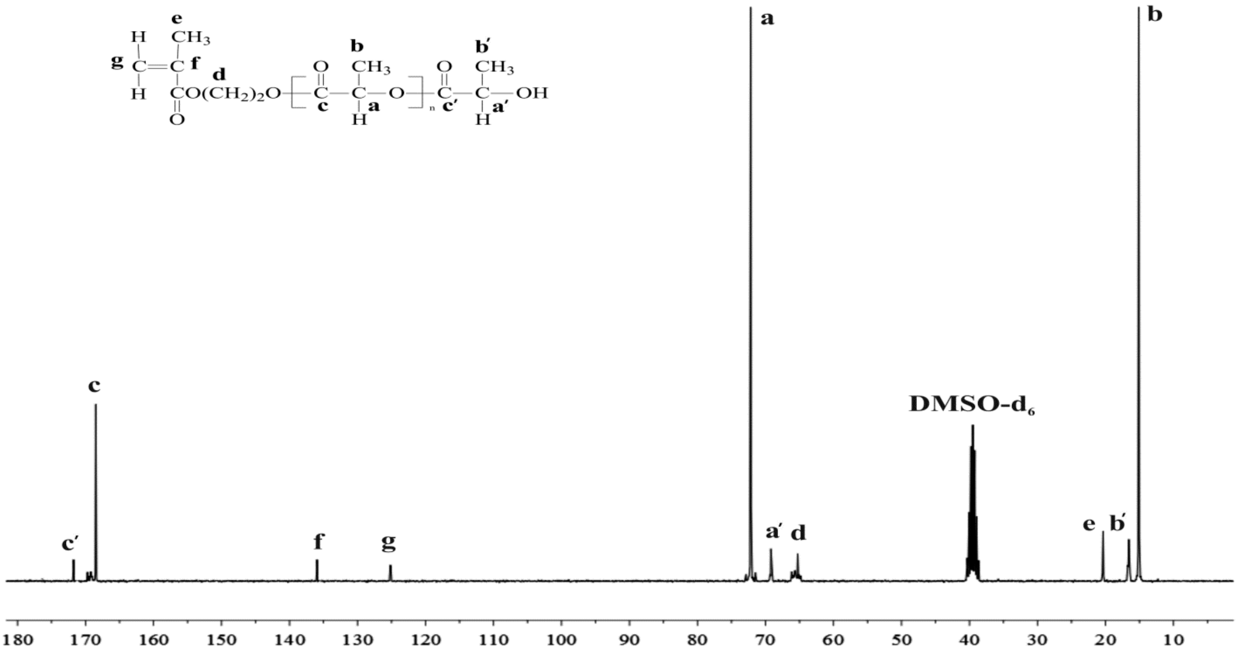

Figure S3. ^{13}C NMR spectrum of the HEMA-PLA100.

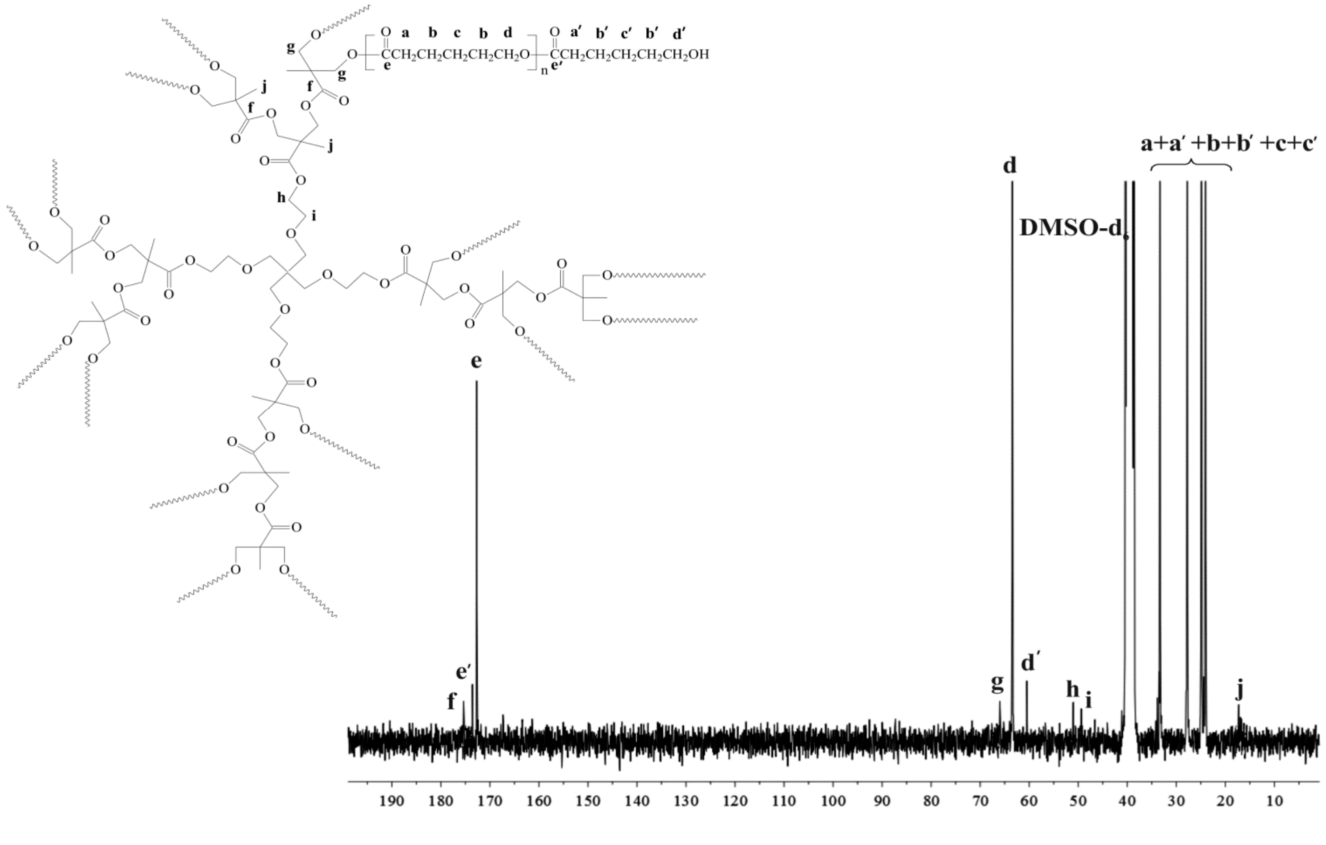

Figure S4. ^{13}C NMR spectrum of the bis-MPA-PCL200.

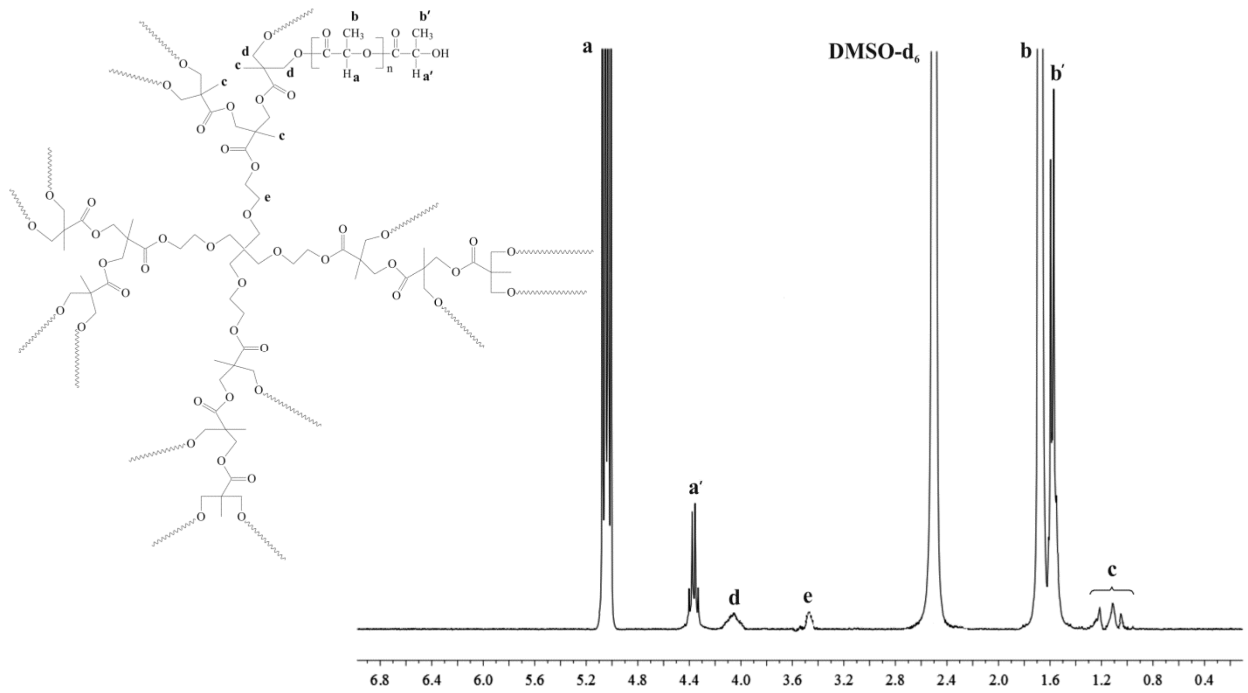

Figure S5. ^{1}H NMR spectrum of the bis-MPA-PLA200.

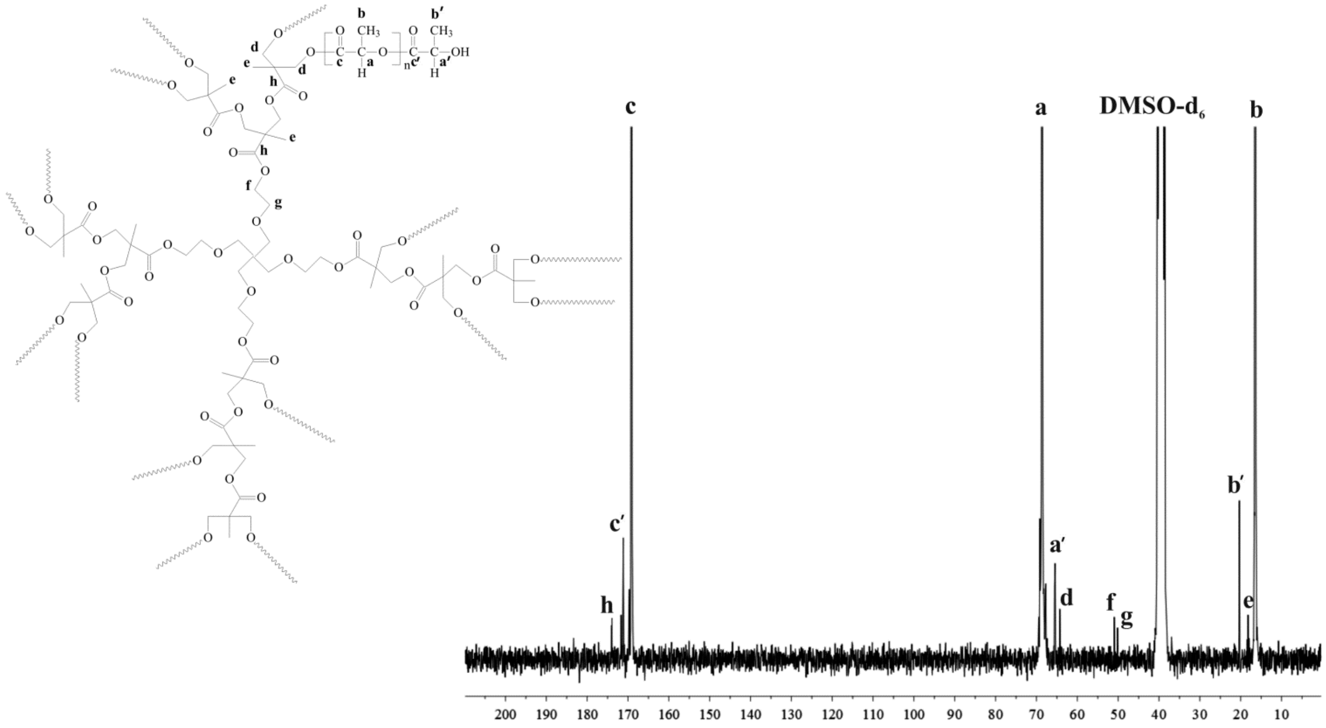

Figure S6. ^{13}C NMR spectrum of the bis-MPA-PLA200.
